# Fat Necrosis Mimicking Local Recurrence With Fluorodeoxyglucose Positron Emission Tomography (FDG-PET) Positivity After Partial Nephrectomy for Renal Cell Carcinoma

**DOI:** 10.7759/cureus.40327

**Published:** 2023-06-12

**Authors:** Toshimitsu Tanaka, Takahiro Yoshida, Yoko Maegawa, Masahiro Nakagawa, Hidefumi Kishikawa

**Affiliations:** 1 Urology, Hyogo Prefectural Nishinomiya Hospital, Nishinomiya, JPN

**Keywords:** laparoscopic partial nephrectomy, carcinomas renal cell, recurrence local, fat necrosis, fdg-pet/ct scan

## Abstract

We report a case of fat necrosis with positive results on fluorodeoxyglucose positron emission tomography (FDG-PET)-CT imaging after partial nephrectomy. A 77-year-old man underwent a partial nephrectomy for a right renal mass. The histopathological results showed clear cell renal cell carcinoma, G1>G2, pT1a. Four and a half years after surgery, a nodule appeared in the retroperitoneal space on CT. FDG-PET CT showed increased uptake in the nodule, indicating local recurrence of carcinoma. A right nephrectomy was performed. The histopathological diagnosis was fat necrosis.

## Introduction

Fat necrosis (FN) is benign inflamed adipose tissue resulting from vascular injury to adipocytes that is usually asymptomatic [1,2]. Abdominal FN is often incidentally identified as a discernible nodule in cross-sectional imaging, typically as a consequence of surgical interventions, traumatic events, or pancreatitis [1,3,4]. Differentiating abdominal FN from malignancies on diagnostic imaging modalities, such as computed tomography (CT) scans and magnetic resonance imaging, can pose difficulties, particularly in cases following abdominal surgeries for malignant tumors [2,4].

Despite enriched adipose tissue in perirenal space, FN after renal surgery is rare. There is only one case report in the literature of FN in perirenal fat after renal surgery for kidney cancer, which mimicked local recurrence [5]. In this report, we present an additional case, which notably is the first such case to be positive in fluorodeoxyglucose positron emission tomography (FDG-PET) CT, posing difficulties in the preoperative diagnosis of FN after renal surgery.

## Case presentation

**Figure 1:**
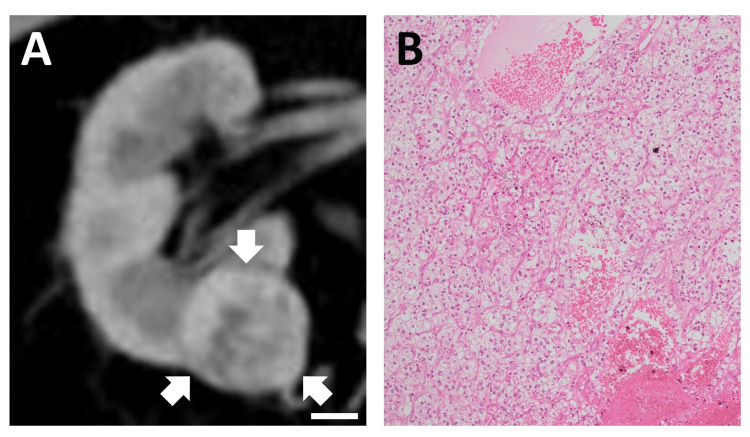
Renal cell carcinoma of the right kidney. (A) Contrast-enhanced CT scan shows a 2-cm nodule on the lower pole of the right kidney (white arrows). Scale bar, 1 cm. (B) Hematoxylin and eosin stained image showing clear cell renal cell carcinoma, G1>G2, pT1a (40× magnification).

A 76-year-old man underwent retroperitoneoscopic partial nephrectomy for right kidney cancer (Figure 1A).

**Figure 2:**
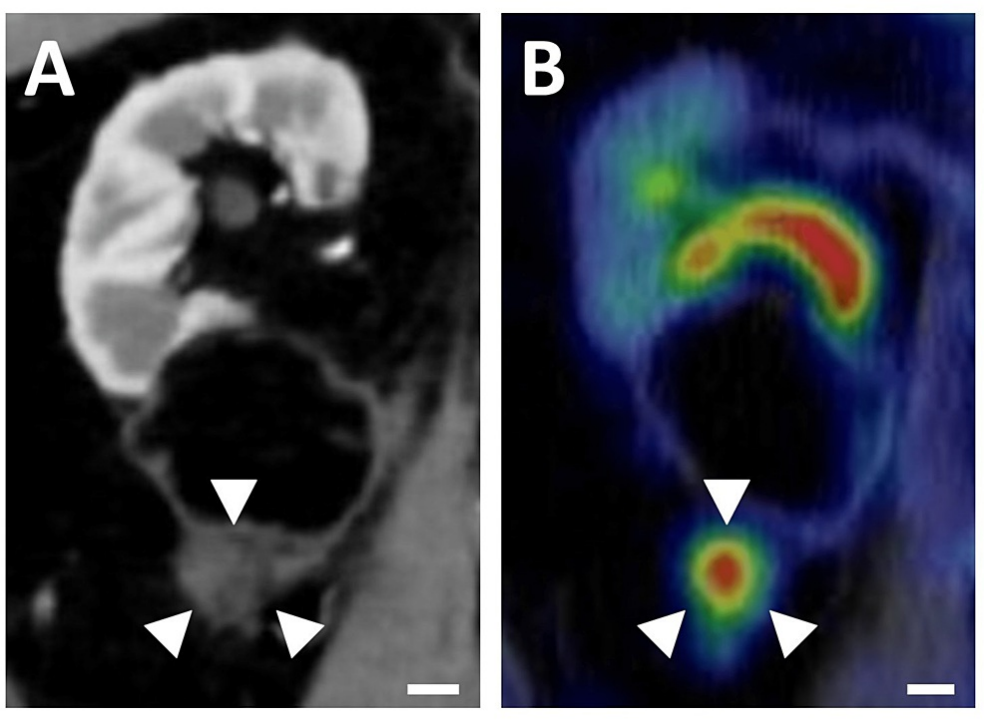
Nodular lesion in the perirenal fat with FDG-PET positivity. (A) Contrast-enhanced CT scan shows a retroperitoneal 2-cm nodule adjacent to the right perirenal fat (white arrowheads). Scale bar, 1 cm. (B) PET-CT exhibited an uptake (SUV-max 5.5) in the same nodule (white arrowheads). Scale bar, 1 cm.

The histopathological results showed clear cell renal cell carcinoma, G1>G2, pT1a (Figure 1B). After 4.5 years of follow-up, an abdominal CT scan revealed a retroperitoneal 2-cm nodule adjacent to the right perirenal fat (Figure 2A), which was not present on imaging 6 months earlier (Figure 4 in Appendices). The nodule showed slight enhancement in the late phase of dynamic contrast-enhanced CT (Figure 5 in Appendices), with an enhancement pattern distinct from that of the renal tumor. FDG PET-CT demonstrated increased uptake (SUV-max 5.5) in the nodule, indicating local recurrence of carcinoma (Figure 2B).

**Figure 3:**
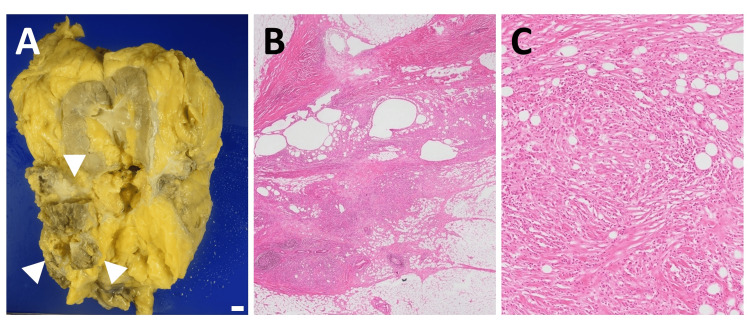
Macroscopic view and histopathological findings of the resected specimen. (A) Formalin-fixed specimen showing a 5-cm firm mass within the perirenal fat (white arrowheads). Scale bar, 1 cm. (B) Hematoxylin and eosin stained image showing a nodular lesion consisting of an inflammatory cell infiltrate with adipocyte size irregularities, fibrosis, and lymph follicle formation (12.5× magnification). (C) Strongly magnified, there are inflammatory cells surrounding the adipocytes, and some foreign-body type multinucleated giant cells are also seen (100× magnification).

**Figure 4:**
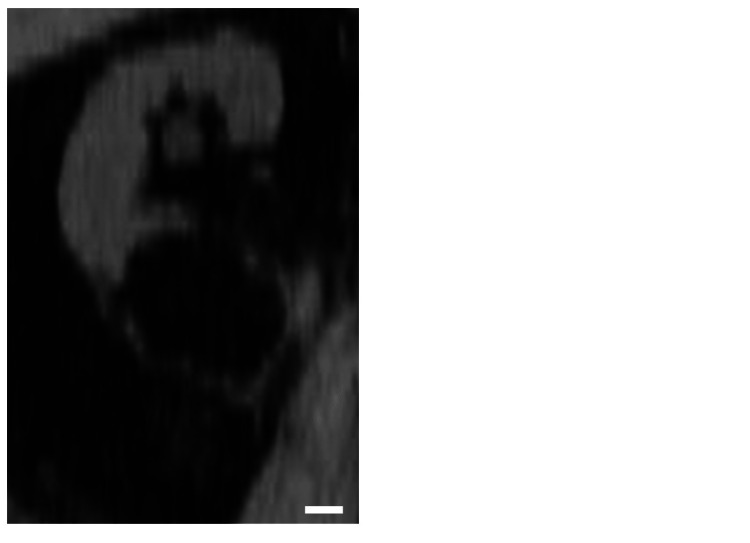
Right kidney six months before the nodule appearance. Plain CT scan shows a low-density area without the nodule in the lower pole of the kidney. Scale bar, 1 cm.

**Figure 5:**
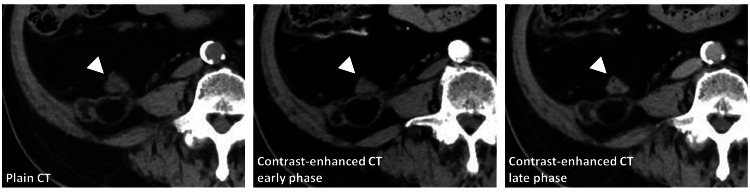
The nodule on a contrast-enhanced CT scan. (A-C) The nodule showed slight enhancement in the late phase of dynamic contrast-enhanced CT (white arrowhead).

Blood tests and urinalysis showed no other abnormal findings than mild anemia (Table 1 in Appendices), indicating the absence of systemic or urinary tract inflammation. Although either needle or open a biopsy was considered, we opted against its implementation due to the limited ability to definitively exclude malignancy, regardless of the biopsy results. Consequently, a decision was made to proceed with radical surgery. Initially, retroperitoneoscopic surgery was attempted. However, due to a high degree of perirenal adhesions, likely resulting from a previous surgery, the procedure had to be converted to open surgery. As a result, the patient underwent open right nephrectomy with excision of the perirenal fat tissue, including the nodule. The resected specimen revealed a 5-cm firm mass located within the perirenal fat (Figure 3A).

Intriguingly, this mass corresponds to the space-occupying lesion, which includes both the nodule and the low-density area above the nodule as indicated in the preoperative CT images. Histopathological analysis revealed adipose tissue with foreign-body type multinucleated giant cells surrounded by inflammatory cells, without any malignant cells. No histopathological differences were found between the nodule and the low-density area above the nodule as indicated in the preoperative CT images. The final pathological diagnosis was FN (Figures 3B, 3C).

## Discussion

FN is a benign inflammatory disease that commonly occurs as a result of vascular injury to adipocytes, such as trauma, surgery, or infection. It is typically found in the breast and subcutaneous tissue, with rare occurrences in other locations [1,2]. FN in perirenal adipose tissue has not been widely reported. In this report, we present the second case in the literature of FN mimicking local recurrence after renal surgery for kidney cancer [5]. Notably, this is the first such case to show positive results in FDG-PET CT, highlighting the diagnostic challenges in differentiating FN in perirenal fat after renal surgery from malignancy using imaging techniques, including FDG-PET CT.

FDG-PET CT has shown remarkable sensitivity in the detection of malignancies, establishing it as a standard procedure for distinguishing malignancies from benign tissue [6]. However, it is important to acknowledge that increased FDG uptake can also be observed in cases of FN, because benign hypermetabolic lesions, such as inflammatory tissue, may exhibit elevated FDG uptake as well [4]. FN can present varying degrees of FDG uptake intensities [4], rendering SUVmax unreliable for definitive diagnosis. Therefore, differentiating between FN and malignancies in FDG PET-CT scans poses an equally challenging task, as exemplified in the present case.

Despite the abundant adipose tissue in the perirenal space, FN in this particular tissue is rare. In a comprehensive review of an institutional FDG-PET CT database, Davidson et al. identified 44 patients with FN following open abdominal surgery, with the omental fat emerging as the most common site of occurrence (43%) [4]. This may be attributed to the rich vascularization of the omentum, which is locally injured during the operation. In contrast, the comparatively diminished vascularity of the perirenal fat may explain the rarity of FN in this space.

## Conclusions

We presented a rare case of FN that was initially suspected to be a local recurrence in a patient who had undergone a partial nephrectomy for kidney cancer. Continued analyses of similar cases will enhance the awareness of this differential diagnosis, thereby avoiding unnecessary surgical interventions or therapies whenever possible.

## Appendices

**Table 1:** Results of preoperative blood test and urianalysis.

			Unit
Blood test	Na	143	nmol/L
	K	4.3	nmol/L
	Cl	107	nmol/L
	Ca	9.5	mg/dL
	Cre	0.9	mg/dL
	T.Bil	0.3	mg/dL
	Alb	4.5	g/dL
	AST	12	U/L
	ALT	15	U/L
	CRP	0.1	mg/dL
	WBC	6370	100/µL
	RBC	439	10,000/µL
	Hb	13.4	g/dL
	Plt	19.2	10,000/µL
Urine sediment	RBC	0-1	/hpf
	WBC	0-1	/hpf
	Bacteria	-	/hpf
